# Correction: Facile preparation of novel quaternary g-C_3_N_4_/Fe_3_O_4_/AgI/Bi_2_S_3_ nanocomposites: magnetically separable visible-light-driven photocatalysts with significantly enhanced activity

**DOI:** 10.1039/d1ra90125g

**Published:** 2021-06-23

**Authors:** Anise Akhundi, Aziz Habibi-Yangjeh

**Affiliations:** Department of Chemistry, Faculty of Science, University of Mohaghegh Ardabili P.O. Box 179 Ardabil Iran ahabibi@uma.ac.ir +98 045 33514701 +98 045 33514702

## Abstract

Correction for ‘Facile preparation of novel quaternary g-C_3_N_4_/Fe_3_O_4_/AgI/Bi_2_S_3_ nanocomposites: magnetically separable visible-light-driven photocatalysts with significantly enhanced activity’ by Anise Akhundi *et al.*, *RSC Adv.*, 2016, **6**, 106572–106583. DOI: 10.1039/C6RA12414C.

The authors regret that incorrect images were used in Fig. 2i (page 106575) and Fig. 3b (page 106576). The correct images are shown below.
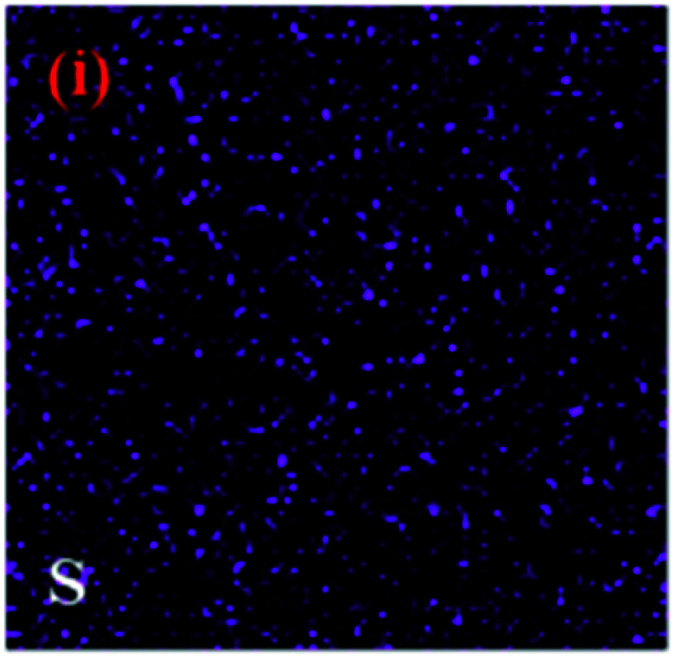


Fig. 2i. EDX mapping of the g-C_3_N_4_/Fe_3_O_4_/AgI/Bi_2_S_3_ (30%) nanocomposite.
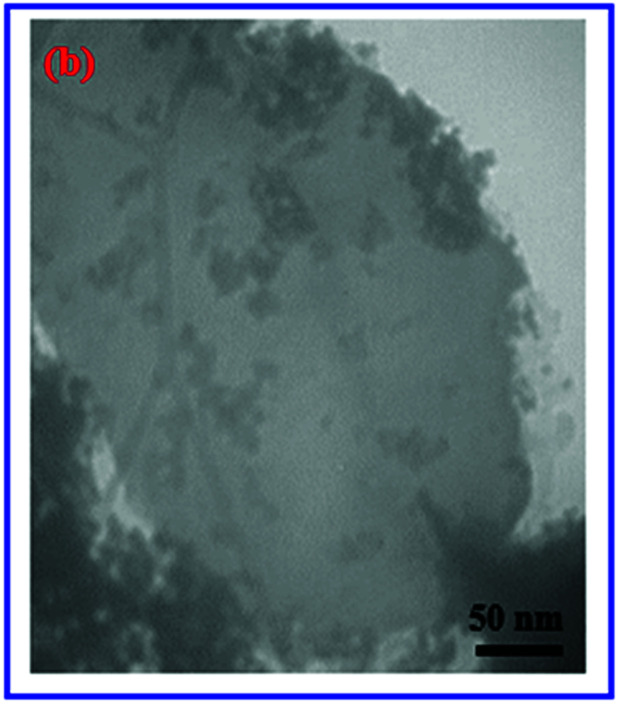


Fig. 3b TEM image of the g-C_3_N_4_/Fe_3_O_4_/AgI/Bi_2_S_3_ (30%) nanocomposite.

The Royal Society of Chemistry apologises for these errors and any consequent inconvenience to authors and readers.

## Supplementary Material

